# Molecular Basis of Calcium-Induced Acidic Shift in Antimicrobial Zinc Sequestration by S100A12

**DOI:** 10.1021/acs.jpcb.5c04464

**Published:** 2025-09-19

**Authors:** Mahil Kothalawala, Shaan Shirazi, Qian Wang, Ahava Collado, Angelo Bongiorno, Rupal Gupta

**Affiliations:** † Department of Chemistry, College of Staten Island, 2009 City University of New York, New York, New York 10314, United States; ‡ Ph.D. Programs in Biochemistry and Chemistry, The Graduate Center of the City University of New York, New York, New York 10016, United States

## Abstract

Antimicrobial protein S100A12 sequesters Zn­(II) via a His_3_Asp motif to inhibit pathogens during infection. Here, UV–vis and NMR spectroscopies and molecular dynamics (MD) simulations are used to gain molecular insight into the Zn­(II) chelation properties of S100A12 under pH conditions relevant to infection and inflammation. UV–vis measurements show that binding of Zn­(II) to apo S100A12 exhibits a sigmoidal dependence with pH, beginning its decline at pH 7.0 and vanishing at pH 4.0. In the Ca­(II)-bound protein, a similar sigmoidal curve is found to exhibit an acidic shift, an effect not attributed to a Ca­(II)-induced p*K*_a_ suppression of the His_3_Asp scaffold. NMR measurements show that upon lowering the pH, resonances exhibit nonlinear migration trends with pH, suggesting the occurrence of several proton binding events, consistent with the sigmoidal pH dependence of Zn­(II) binding. Analysis of the NMR chemical shifts versus pH shows that both apo- and Ca­(II)-bound protein undergo conformational changes exhibiting spatial correlations, which are dispersed across the polypeptide in the apo protein and confined to discrete regions in Ca­(II)-S100A12. MD simulations show the formation of a strong salt bridge within the His_3_Asp scaffold of the apo protein upon protonation of Zn­(II)-ligating histidines. Geometric constraints imposed by Ca­(II) in the Ca­(II)-bound protein hinder the formation of similar salt bridges until protonation of a histidine residue external to the His_3_Asp and near Ca­(II) takes place. Overall, our experimental and computational results support a scenario where protonation of the His_3_Asp motif triggers the loss of Zn­(II) binding and yields protonated histidine residues prone to form stable salt bridges. In Ca­(II)-S100A12, formation of stable salt bridges at pH 7 is hindered compared to the apo form, thus extending Zn­(II)-binding affinity to lower pH.

## Graphical abstract

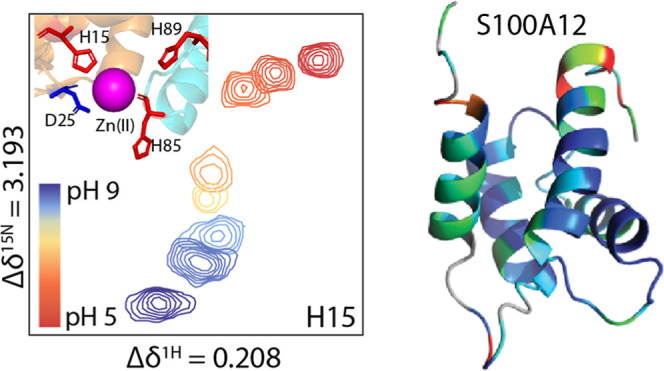

## 1 Introduction

Antimicrobial S100 proteins such as the S100A12 homodimer and S100A8/A9 heterodimer are secreted by human immune cells to sequester transition metal nutrients, particularly Zn­(II), thereby preventing proliferation of pathogenic cells and curtailing infection.^1,2^ All S100 proteins bear two calcium-binding EF loops, and transition metal chelation by these proteins has been shown to be enhanced upon Ca­(II) binding (*K*_d_ ∼ nM at neutral pH).^1,3^ Structural characterization of various apo- and Ca­(II)-loaded S100 proteins has demonstrated that upon Ca­(II) binding, these proteins undergo a major structural rearrangement, consisting mainly of the reorientation of helix 3.^4,5^ It remains unclear how this structural change, or simply the presence of Ca­(II) in the EF loops, influences the transition metal chelation by S100A12. In particular, Zn­(II)-binding affinity, known to diminish at acidic pH in the apo protein, is rescued in the presence of Ca­(II) for both S100A12 and S100A8/A9.^6,7^ The origin of this Ca­(II)-mediated effect is not understood. Here, we combine UV–vis and NMR experiments and molecular dynamics (MD) simulations to gain insight into the origin of this unclear phenomenon.

Antimicrobial S100 proteins are produced by immune cells such as neutrophils during infection.^2^ For a viable immune response, these proteins must withstand biological conditions relevant to pathogenesis. For example, acidic pH regimes are observed at sites of infection such as airways in the cystic fibrosis lung and the gut during inflammatory bowel disease.^8,9^ Indeed, neutrophils are activated under extracellular acidosis,^10,11^ suggesting a correlation between acidic pH regimes and initiation of the immune response. In the context of metal storage, pH-dependent retention abilities of Fe­(III) binding by transferrin and lactoferrin have been documented. For transferrin, the Fe­(III) chelating affinity reduces below pH 7.4, enabling the release of ferric ions,^12^ while lactoferrin withholds its ferric chelation affinity at sub neutral pH conditions.^13^ Interestingly, pathogens like *Staphylococcus aureus* are believed to acidify their environment to obtain Fe­(III) from transferrin, suggestive of a combat strategy utilized by pathogens to spread infection.^14^ With these strategies undertaken by both the host and the pathogens, in this work, we evaluate the metal chelation response of S100A12 under chemically diverse conditions relevant during infection and inflammation.

Transition metal sequestration by antimicrobial S100 proteins takes place at their dimeric interface at a His_3_Asp scaffold composed of residues from both monomers. In the case of the homodimeric S100A12, the two symmetrical His_3_Asp motifs are composed of H85 and H89 belonging to one subunit and H15 and D25 from the second subunit.^15,16^ For the heterodimeric S100A8/A9, the transition metal binding sites are asymmetrical, consisting of a His_3_Asp scaffold similar to S100A12, and a His_6_ motif.^17^ Among the two Ca­(II)-binding loops, EF I is located at the N-terminus, tethered by helices 1 and 2. This S100-specific 14 amino acid long loop is composed of residues 18–31 in S100A12. The EF loop at the C-terminal (EF II) is located between helices 3 and 4, and this conical motif is found in all Ca­(II)-binding proteins (residues 61–72). The main difference between the EF I and EF II loops pertains to the coordination environment of Ca­(II) ions. Canonical EF loops, like the EF II loop of S100 proteins, are composed of acidic amino acids binding to Ca­(II) via side chain carboxyl groups. In contrast, EF I loops tether Ca­(II) via backbone carbonyl ligands, and the loop bears several basic residues.^18^ While distinct, the EF loops in S100A12 are dynamically coupled, suggesting their cooperativity.^19,20^ We have shown that the E31A variant of S100A12, which binds to Ca­(II) only at the EF II loop, bears a structure similar to the wildtype protein in the presence of Ca­(II).^20^ These studies also demonstrated that Ca­(II) binding to the EF II loop dictates the overall architecture of the protein, with EF I playing a limited role toward introducing structural perturbations. Being in the vicinity of H15, which binds to Zn­(II)—and bearing residue D25, the EF I loop is expected to influence Zn­(II) chelation by the protein.

Recently, we have shown that Ca­(II) can rescue Zn­(II) chelation by S100A12 at acidic pH conditions, without altering the p*K*_a_ of the His_3_Asp scaffold.^6^ Understanding the molecular mechanisms underlying Ca­(II)-mediated Zn­(II) sequestration of S100A12 is critical for developing a working model of the immune response and how the human body combats infection. Here, we use UV–vis and NMR spectroscopy and MD simulations to gain molecular insight into the role played by Ca­(II) in expanding the viable metal sequestration by S100A12. The article is organized as follows. In Section 2, we provide technical details about samples and experiments, data analysis, and MD simulations. In Section 3.1, we discuss the results of UV–vis experiments carried out to probe the Zn­(II)-binding affinity of S100A12 at pH ranging from 4.0 to 8.0. Results about the pH response of Zn­(II) chelation of the E31A mutant of S100A12 are also reported in this section. In Section 3.2, we report and discuss the results of NMR measurements of the apo- and Ca­(II)-bound forms of S100A12 carried out to probe the structural changes occurring in the proteins throughout the aforementioned pH interval. In Section 3.3, we report on the results of MD simulations and articulate a picture explaining the collection of our experimental results. Conclusions are listed in Section 4.

## 2 Methods

### 2.1 Sample Preparation and Experimental Details

Recombinant human S100A12 protein, both uniformly ^15^N-labeled and unlabeled, was overexpressed in *Escherichia coli* and purified according to previously established protocols.^21^ UV–vis spectroscopy measurements were conducted to monitor the pH-dependent binding affinity of Zn (II) ions to S100A12. Samples were prepared at a final protein concentration of 0.15 mM in a mixed buffer system comprising 20 mM MES:TRIS:CHES and 150 mM NaCl. The pH of the buffer was adjusted across the range of 4.0 to 8.0 using dilute HCl or NaOH. For Ca­(II)-S100A12, 20 equiv of Ca­(II) was added to the protein solution. Co­(II), used as a spectroscopically active surrogate for Zn­(II) due to its comparable coordination chemistry, was freshly prepared to prevent oxidation. The Co­(II)-S100A12 complex was monitored by measuring the absorbance at 556 nm. All absorbance data were baseline-corrected and normalized prior to analysis.

For NMR spectroscopy, uniformly ^15^N-labeled S100A12 samples were prepared at a final concentration of 0.3 mM. For pH ranges of 5.0 to 9.0, the protein was buffered in 20 mM MES:TRIS:CHES with 150 mM NaCl, whereas samples at pH 4.0 to 5.0 were prepared in 20 mM sodium acetate and 20 mM acetic acid with 150 mM NaCl. All NMR samples contained 10% D_2_O and 200 μM 4,4-dimethyl-4-silapentane-1-sulfonic acid (DSS) as an internal reference. The pH was adjusted by using dilute NaOH or HCl. 2D ^1^H–^15^N heteronuclear single quantum coherence (HSQC) spectra were acquired on a 600 MHz Varian NMR spectrometer equipped with an HCN cryoprobe. The Larmor frequencies were 599.93 MHz for ^1^H and 60.79 MHz for ^15^N, and the sample temperature was maintained at 25 °C. Spectra were processed by using NMRPipe and analyzed with Sparky. Linear forward prediction to double the original data points, followed by zero-filling to twice the total number of points, was applied. Apodization employed 30°- and 60°-shifted sine-bell functions along with water suppression in both dimensions. A total of 10 HSQC spectra were collected for the apo-S100A12 protein across the pH range of 5.0 to 9.0. For the Ca­(II)-bound form, 16 spectra were recorded over the pH range of 4.0 to 9.0. Chemical shift perturbations (CSPs) were calculated for each residue. The standard deviation of the CSPs for each residue was calculated to assess the chemical shift of each residue across the pH interval.

### 2.2 Single Value Decomposition and Statistical Analysis of the NMR Data

The chemical shifts of the backbone ^15^N atoms of both the apo- and Ca­(II)-bound forms of S100A12 are ordered as a *m* × *n* matrix ***D***, with columns containing the chemical shifts assigned to the *m* = 91 residues of the two protein units obtained at the pH values between 4.0 and 9.0. The matrix is factorized (according to compact single value decomposition (SVD)) as ***D*** = ***UWV*** ^*T*^, where ***U*** is a *m* × *n* matrix of orthogonal (chemical shift) columns ***u***_*i*_, ***W*** is a diagonal *n* × *n* matrix of singular values sorted in decreasing order, and ***V*** is a *n* × *n* matrix whose columns ***v***_*i*_ contains the weights of each chemical shift vector ***u***_*i*_ assigned to the singular values *w*_*i*_. According to SVD, each matrix element of ***D*** can be expressed in terms of its singular values as $D_{ij}=\sum_{k=1}^{n}w_{k}u_{ki}v_{jk}$ . Here, following Wright et al.,^22^ we consider a singular value as significant and the corresponding component as non-noise when both of the following conditions are satisfied: (i) the singular value is larger than 1 and (ii) the coordinates of the corresponding autovector ***v***_*i*_ exhibit a smooth shape (i.e., high autocorrelation). Using these two criteria, we can state that the SVD analysis of the NMR data shows the occurrence of about 3–4 non-noise components in the case of both the apo- and Ca­(II)-loaded proteins.

We also carried out a statistical analysis of the chemical shifts of the backbone ^15^N atoms obtained under various pH conditions. In particular, given the *m* row vectors ***d***_*i*_ (of the matrix ***D***) containing the chemical shifts of the ^15^N atom in residue *i* (running from 1 to 91), first we calculated the average values, ${\mathrm{d̅}}_{i}$ , and standard deviations, δ_*i*_ (this latter quantity provides an estimate of the overall sensitivity of a residue to pH conditions), and then we calculated the correlation coefficients: $C_{ij}=\left[\sum_{k=1}^{n}\left(d_{ik}-{\mathrm{d̅}}_{i}\right)\left(d_{jk}-{\mathrm{d̅}}_{j}\right)\right]$ /δ_*i*_/δ_*j*_.

### 2.3 MD Simulations

MD simulations were carried out using GROMACS v.2024.^23^ Our all-atom explicit-solvent MD simulations employed the TIP3P water model^24^ and the ff99SB*-ILDN force field.^25,26^ Our systems encompass a homodimer S100A12 protein in a periodic cubic box (with dimensions such that a minimum distance of 3 nm separates the protein replicas), surrounded by water molecules and a number of chlorine and sodium ions to neutralize the charge carried by the metal-binding protein and obtain a 0.15 M NaCl aqueous solution. Our systems (containing up 23,320 atoms) are equilibrated using a standard protocol, followed by a production MD run at 298 K and 1 bar spanning 2 μs. The isobaric–isothermal MD simulations rely on the Berendsen thermostat and Parrinello–Rahman barostat methods to control the temperature and pressure, respectively. Equations of motion are integrated by using the leapfrog algorithm with a time step of 2 fs; holonomic constraints are imposed on bonds with H. The (Particle Mesh) Ewald method is used to calculate electrostatic interactions, with a cutoff radius of 10 Å to truncate short-range interactions.

Atomistic models of S100A12 homodimers were constructed based on the PDB files 1GQM (calcium-bound, chains G and H) and 2WCF (calcium-free, chains A and B). The E31A variant of S100A12 was obtained from PDB file 1GQM, after replacing the amino acid E31 with alanine. In the case of calcium-free apo protein and the E31A protein hosting Ca­(II) ions in the EF I loops, we run a 2 μs long MD run mimicking the proteins at low pH conditions, i.e., with all histidine residues (H6, H15, H23, H85, H87, and H89) in the protonated state. In the case of the Ca­(II)-loaded protein, we consider H15, H85, H87, and H89 in their protonated state and the following four protonation states for H6 and H23: (neutral, neutral), (protonated, neutral), (neutral, protonated), and (protonated, protonated), where the first case mimics the protein at pH 7,^6^ whereas the remaining three configurations are intended to simulate the protein at increasingly lower pH. For each one of these cases, we run a MD simulation for 2 μs. To determine the occurrence of salt bridges involving the aspartic acid residue D25 and the neighboring H15, H23, and H85 residues, from the MD trajectories, we extracted the minimal distance between the O atoms of the carboxyl group in D25 and either the N_δ_ or N_ε_ atoms of the histidine residues. To quantify (qualitatively) the strength of a salt bridge bond between D25 and a histidine residue, we compute the occurrence “frequency”, defined as the number of configurations over the total spanned by a MD run in which the salt bridge occurred with a distance less than 3 Å.

## 3 Results and Discussion

### 3.1 Zn­(II) Binding to S100A12 versus pH: UV–Vis Measurements Using Co­(II) as Surrogate

The Zn­(II)-bound state of S100A12 is spectroscopically silent, with no detectable UV–vis transition. Sharing a similar coordination chemistry, Co­(II) has been shown to bind to the His_3_Asp Zn­(II) coordinating motif in S100A12.^1^ Furthermore, the Co­(II)-S100A12 complex exhibits a well-defined UV–vis signature at an λ_max_ of 556 nm. Thus, here we use Co­(II), instead of Zn­(II), to probe the metal chelation efficacy of S100A12. To quantify the effect of pH on metal binding, we carried out pH-dependent UV–vis measurements of Co­(II)-S100A12 and recorded the absorbance at 556 nm at the various pH conditions. At neutral pH, S100A12 exhibits a strong nanomolar affinity toward Co­(II) (or Zn­(II)). Thus, near complete Co­(II) binding is achieved at a larger pH using a micromolar concentration of the protein (Section 2.1). The absorbance at 556 nm under these conditions is used as a reference to quantify the fractional occupancy of the His_3_Asp motifs with Co­(II) at lower pH values.^1,6^ In these experiments, the pH of the Co­(II)-S100A12 complex, generated by adding 1 equiv of Co­(II) to S100A12 (Section 2.1), was varied in the absence and presence of Ca­(II) to probe the contribution of Ca­(II) to the Zn­(II) affinity of the protein. The results of these experiments are listed in Figure 1.

**Figure 1:**
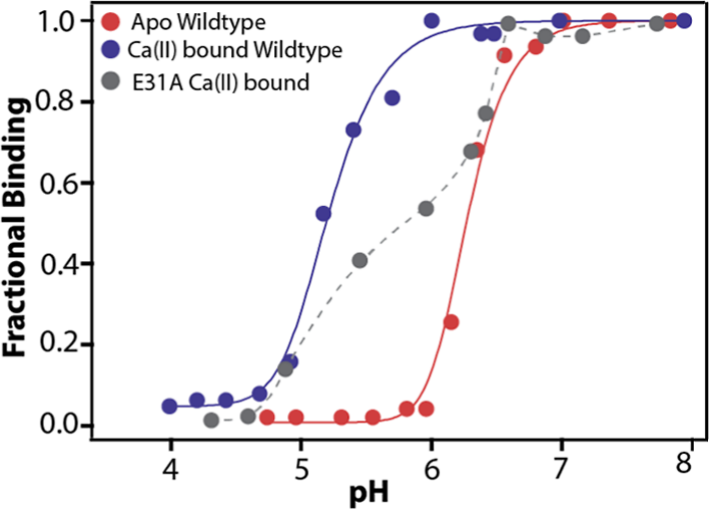
pH dependence of Co­(II) binding to this His_3_Asp motif of apo wildtype (• (red)), Ca­(II)-bound wildtype (• (blue)), and Ca­(II)-bound E31A (• (gray)) S100A12 monitored via electronic absorption transition of Co­(II)-S100A12 at 556 nm. Co­(II) is used as a surrogate to Zn­(II) binding to the His_3_Asp motif. The solid traces are fit to experimental data assuming polyprotic titration of four protons with identical p*K*_a_ values. By “fractional binding”, we refer to the quantity *A*_λmax_[pH]/*A*_λmax_[pH = 8], where *A*_λmax_[pH] is the absorbance intensity at 556 nm measured at a particular pH.

UV–vis absorption intensity at 556 nm remains constant for decreasing the pH from 9 to 7 (Figure 1), suggesting that protonation of ionizable groups in this pH range does not influence Co­(II) (Zn­(II)) binding to both apo- and Ca­(II)-bound forms of S100A12. At pH 7.0, the apo protein begins to lose its Co­(II)-binding affinity (Figure 1), as derived from the gradual loss in the peak intensity at 556 nm, and at pH 6.0, no detectable absorbance at 556 nm indicates near-complete loss of Co­(II) bound to the apo protein. Figure 1 shows that the Ca­(II)-loaded protein exhibits greater tolerance toward pH, retaining strong Co­(II) binding until a pH of 6.0. Upon further lowering the pH, Ca­(II)-S100A12 shows a decline in Co­(II) binding very similar in shape to that of the apo form. Numerical simulations assuming protonation of a single ionizable group cannot reproduce the observed sigmoidal curves in Figure 1 (Figure S1), and an acceptable fit to the data is only achieved by including 3 or 4 protonation events in both the apo- and Ca­(II)-loaded protein. Overall, the data in Figure 1 underscore two important points. First, the apo- and Ca­(II)-bound states of S100A12 exhibit similar sigmoidal responses for the Co­(II)-binding affinity versus pH, indicating that 3–4 proton binding events are responsible for the complete loss of metal binding upon lowering the pH. Second, the Ca­(II)-bound protein withholds Co­(II) tightly up to a pH of about 6.0, whereas the apo form loses most of its bound metal under similar conditions.

To gain further insight, we utilize the previously characterized E31A variant of S100A12.^20^ The E31A mutation eliminates Ca­(II) binding to the EF I loop without influencing binding to the EF II loop. We remark that our recent NMR studies have shown that the overall structure of Ca­(II)-E31A is very similar to that of Ca­(II)-loaded wildtype protein, demonstrating that the conformational rearrangement in S100A12 upon Ca­(II) binding is driven by the EF II loop.^20^ Therefore, the E31A variant is an optimal candidate to evaluate the effect of (lack of) Ca­(II) in the EF I loop on the pH-dependent Co­(II) binding of Ca­(II)-S100A12. In particular, our UV–vis experiments on the apo- and Ca­(II)-loaded states of the E31A variant in the presence of Co­(II) show that the apo form exhibits a pH dependence very similar to that of the wild-type protein (data not shown). Upon Ca­(II) binding to the EF II loop of the E31A variant, the viable pH range is extended, and interestingly, the pH response of the Ca­(II)-loaded mutant lies in between the apo- and Ca­(II)-bound wildtype proteins (Figure 1). Overall, these results suggest that both EF loops contribute toward increasing the Co­(II)/Zn­(II)-binding affinity of the protein under acidic conditions.

### 3.2 pH-Induced Conformational Changes in S100A12: NMR Studies

To gain insight into the structural changes triggered by protonation to S100A12, we carried out ^1^H–^15^N HSQC measurements on both the apo- and Ca­(II)-bound proteins under different pH conditions by varying the pH between 4.0 and 9.0. Within this pH interval, NMR spectra of both proteins consist of well-dispersed resonances, indicative of proteins with congruent architecture, thereby showing no signs of denaturation. The NMR spectra of the apo protein collected between pH 5.0 and 9.0 are shown in Figure 2. Based on previously reported resonance assignments,^21^ we identified 67 out of 91 resonances in these spectra. Most resonances undergo fast exchange with gradual perturbation of their chemical shifts in this pH range. Residues G22, K48, K50, G59, T88, and K90 show well-resolved resonances between pH values of 5–7. At higher pH values, their cross-peak intensities are attenuated beyond detection, presumably due to pH-induced intermediate exchange under basic conditions. NMR resonances of a few residues, such as E91, exhibit a near-linear dependence on pH, suggesting the influence of single proton binding. Interestingly, however, the NMR signals of the majority of the residues exhibit nonlinear migration patterns, suggestive of transition between multiple conformations (Figure 2). Following the approach discussed in ref 27, upon ligand (L) binding to a protein, fast exchange between ligand free and ligand bound states yields chemical shifts that are the result of a weighted average of all existing conformations. For a two-site exchange corresponding to a conversion from ligand free to single ligand bound conformation (P + L ⇋ PL), a linear migration of chemical shift is observed from state P to PL when a ligand is titrated to a free protein. In our pH-dependent experiments, [H^+^] is the ligand, and its binding to an ionizable amino acid yields a proton bound conformation (S100A12 + [H^+^] ⇋ S100A12­[H^+^]). As demonstrated by others,^22^ nonlinear migration of chemical shifts during ligand titration suggests the occurrence of multiple binding events (P + *n*L ⇋ PL + PL_2_ + PL_3_···PL_*n*–1_ + PL_*n*_). Based on this, the pH-induced nonlinear patterns of the measured chemical shifts suggest binding of two or more protons. This observation is consistent with the analysis of our UV–vis measurements in Figure 1, also indicating the occurrence of multiple proton binding events.

**Figure 2:**
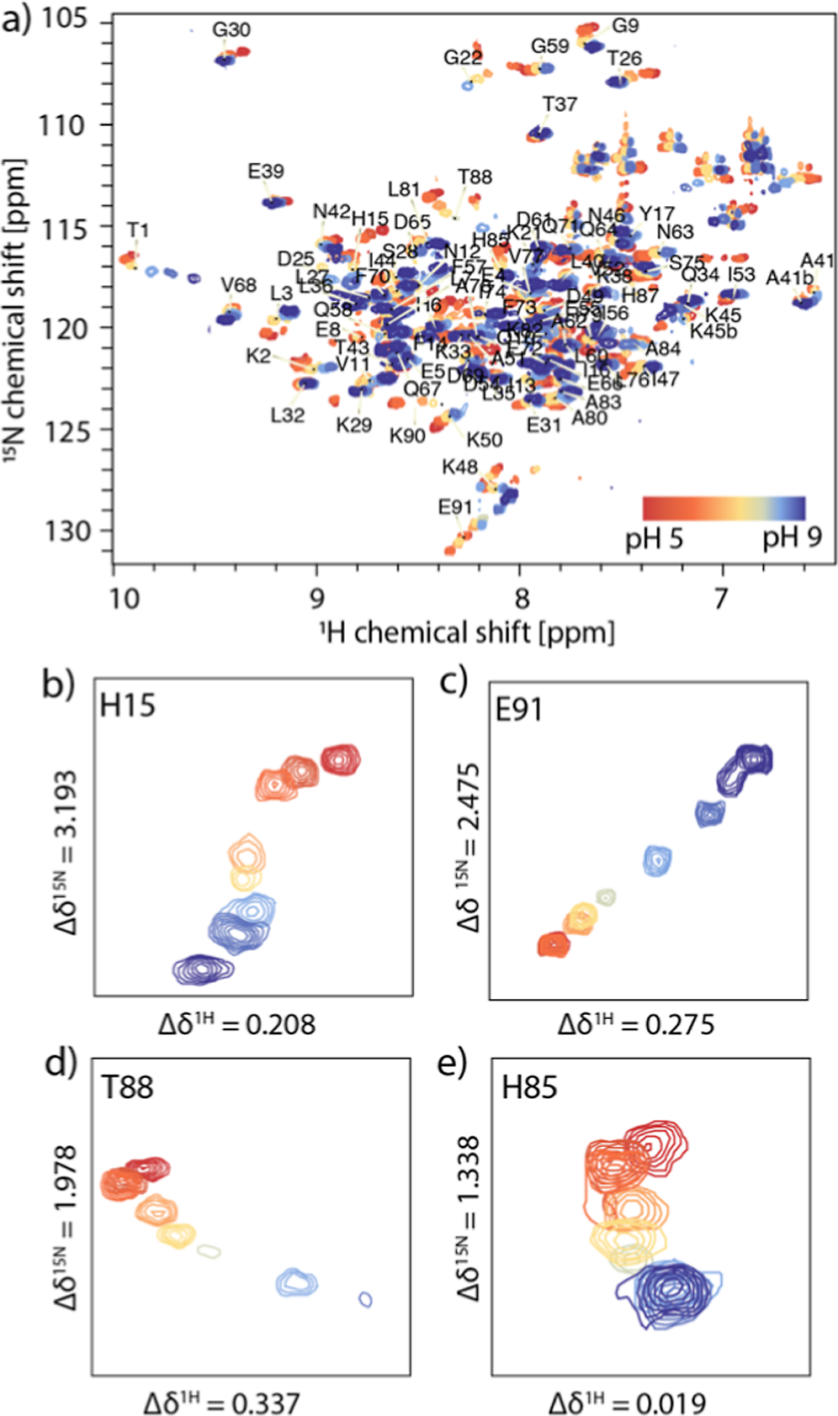
(a) pH effect on conformation of apo S100A12 monitored using ten H–^15^N HSQC correlation spectra of apo S100A12 in the range of pH 5.0–9.0 at 14.1 T and 25 °C. (b–e) Expansions demonstrating migration of resonances for select residues with pH.

We carried out an SVD analysis of the two-dimensional data set consisting of NMR chemical shifts of the residues versus pH values (Section 2). Non-noise components, which are indicative of key events driven by proton concentration, were evaluated based on the magnitude of the singular values and the autocorrelation (based on the smooth shape) of the ν_*i*_ vectors.^28,29^ Our SVD analysis suggests that 3–4 components have significantly larger singular values (Figure 3), with their corresponding ν_*i*_ vectors exhibiting a high degree of autocorrelation (Figure 3, inset). Based on this, the presence of three non-noise components can be attributed to proton-driven conformational changes, consistent with our UV–vis and NMR experiments (Figures 1 and 2). This suggests a correspondence between the detected protonatable sites modulating metal binding and the conformational change monitored via HSQC spectra in the same pH range.

**Figure 3:**
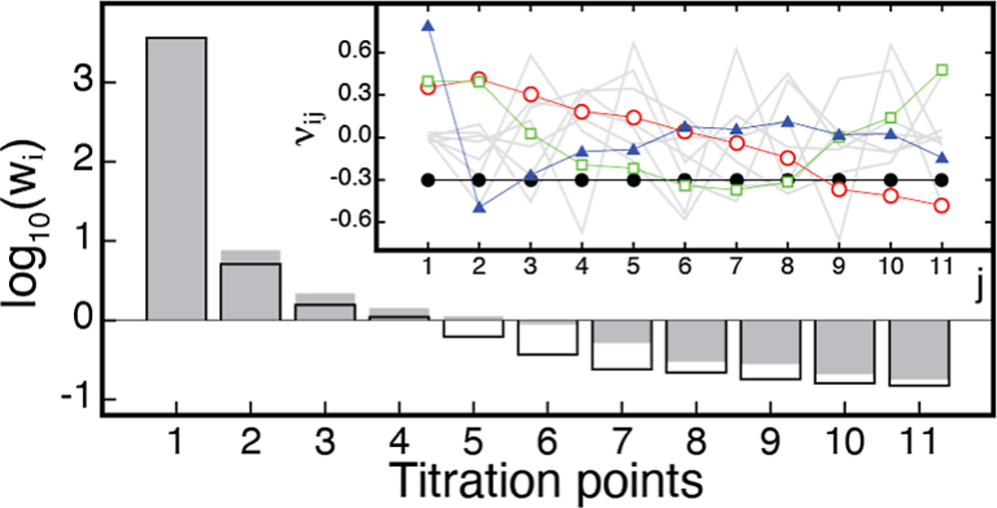
Singular values (sorted in decreasing order and plotted in logarithmic scale) of the *m* × *n* matrix ***D*** containing the chemical shifts of the backbone ^15^N atoms of the *m* = 91 residues measured at *n* different titration points (pH values ranging from 4 to 10) for the apo (gray-filled histograms) and Ca­(II)-bound (empty histograms) forms of S100A12. Inset, ***v***_*i*_ vectors versus titration points. The first four vectors showing a smooth shape are shown in black (1st), red (2nd), green (3rd), and blue (4th) colors, whereas the rest of the (noise) components are shown using light gray lines.

Figure 4a shows residue-wise standard deviations of the ^15^N^H^ chemical shifts (_^15^N_δ^stdev^) of the apo protein at pH values from 4.0 to 9.0 (Section 2). Most residues show high pH susceptibility, with H15 exhibiting _^15^N_δ^stdev^ of 1.1 ppm. In addition to H15, E91 and T88 also exhibit large CSPs, indicative of significant pH-dependent conformational changes. The overall residue-wise trend in the standard deviations is gradual, indicating that the entire protein backbone is pliable to pH changes, lacking discrete proton-mediated processes. To further evaluate if the conformational changes observed by any subset of residues are correlated, we calculated the Pearson correlation coefficient (PCC) between the CSPs of the residues (Figure 4b). The analysis shows high degree of correlation, suggesting that protonatable groups impart global conformational changes throughout the apo protein. In particular, residues in helix 1, hinge, helix 4, and C terminal domain (CTD) exhibit large chemical shift changes (0.25 ppm) and are highly correlated to each other (Figure 4b, pink boxes).

**Figure 4:**
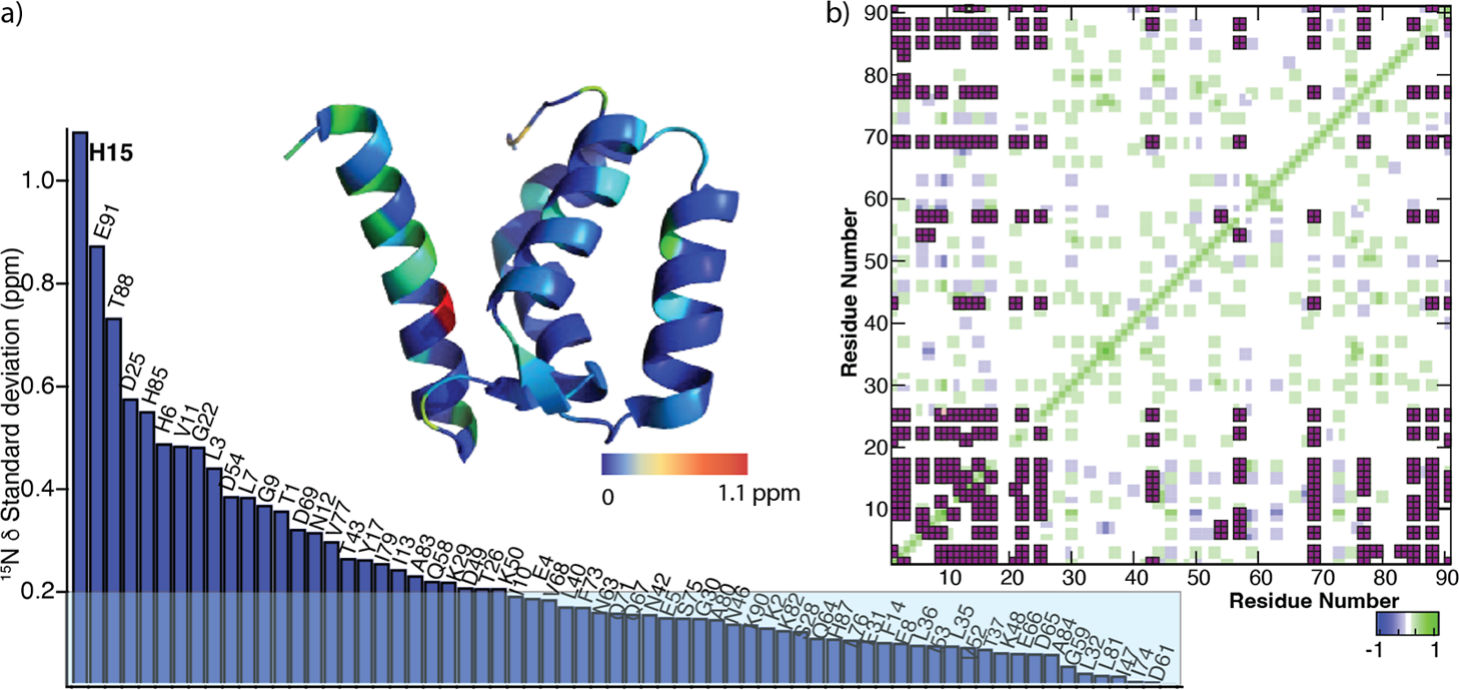
(a) Standard deviations of ^15^N^H^ chemical shift migration during pH titration of the apo protein sorted in decreasing order of magnitude. (b) Residue-wise correlation between the observed chemical shift changes during the pH titration. Only correlations with PCC ≥ |0.9| are shown. The standard deviations in ^15^N^H^ chemical shifts are mapped on to the protein structure (only the monomeric chain is shown for the sake of clarity).

^1^H–^15^N HSQC spectra of Ca­(II)-S100A12 collected at pH values ranging from 4.0 to 9.0 are shown in Figure 5. We assigned the resonances of 76 out of 91 residues of the protein. For all 16 spectra collected in this pH range, no signals associated with the apo form were detected. As for the apo protein, most of the resonances underwent nonlinear migration as proton concentration was varied, suggesting fast exchange processes comprising multiple proton transfer events (Figure 5). Several residues exhibited exchange in the intermediate regime, as evident from the loss of their signal intensities beyond detection as the proton concentration was varied. However, while residues in the apo protein underwent intermediate exchange only at high pH, in the Ca­(II)-bound form, low pH conditions also triggered exchange interactions and signal loss. In particular, regions in helix 1, hinge, and the CTD exhibited signal loss at high pH, while selected residues in the EF-I (H15, G22, and S28) and EF-II loop (V68 and D69) showed loss in signal at low pH. This observation is consistent with our previous study reporting intermediate exchange observed in the EF I loop at sub neutral pH conditions.^19^

**Figure 5:**
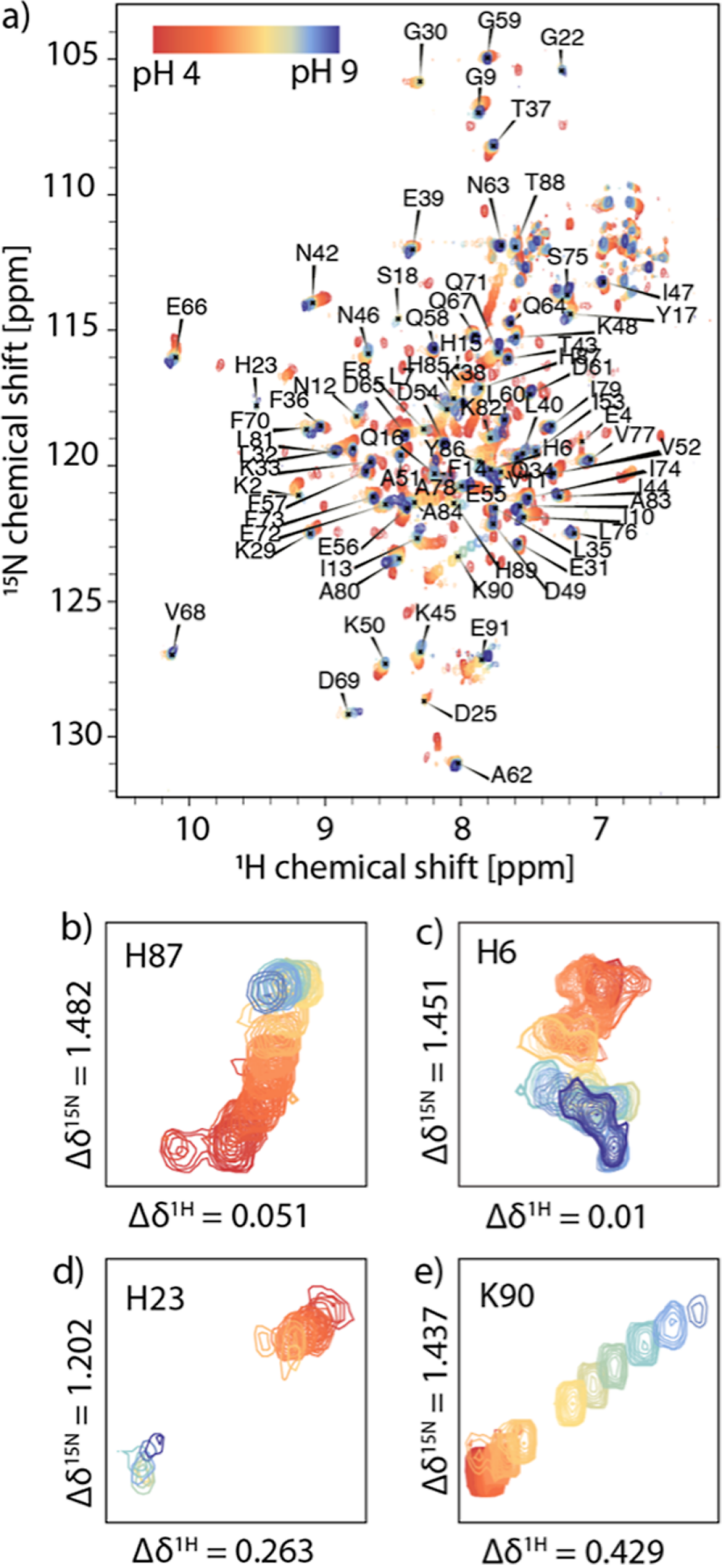
(a) pH response of Ca­(II)-loaded S100A12. Sixteen ^1^H–^15^N HSQC correlation spectra were collected in the range of 4.0–9.0 at 14.1 T and 25 °C. (b–e) Expansions showing migration of resonances for select residues.

Three non-noise components can be identified from SVD analysis of the raw chemical shifts from the NMR titrations based on their large singular values (Figure 3). In comparison to the apo protein, however, beyond the three major components, the singular values of the remaining constituents diminish dramatically, suggesting discrete conformational speciation. Lastly, the presence of three primary proton-mediated events is consistent with the UV–vis measurements, suggesting that proton-driven conformational changes probed by the HSQC spectra manifest the pH-induced metal binding propensity.

Figure 6a shows site-specific _^15^N_δ^stdev^, showing residues undergoing major conformational changes during pH titration. The behavior of the Ca­(II)-loaded form is clearly distinct from the apo protein in two ways. First, the overall magnitude of _^15^N_δ^stdev^ in the presence of Ca­(II) is significantly smaller than that of the apo protein (maximum _^15^N_δ^stdev^ of 0.6 ppm in Ca­(II) versus 1.1 ppm in the apo form). This suggests that Ca­(II) binding affords improved conformational resistance toward pH changes. Second, while the apo protein experienced continuous fluctuations throughout the polypeptide, in the presence of Ca­(II) six residues underwent the most drastic conformational changes. These six residues, namely, H87, H6, H23, K90, H85, and L7, represent the epicenter of the primary conformational changes. The rest of the residues undergo minor conformational changes, as evident from their small CSPs. The PCC analysis shows a high degree of correlation between the observed perturbations among all residues (Figure 6b). This suggests that while most residues undergo minor conformational changes, these are driven by the protonation events leading to major conformational changes at the primary sites. The correlated residues in the PCC analysis undergoing significant _^15^N_δ^stdev^ (0.25 ppm) are localized in helix 1 and CTD as opposed to the apoprotein, where the correlated region also included the hinge and helix 4. This suggests that residues in hinge and helix 4, while susceptible in the apo protein, are shielded toward pH-induced perturbations in the presence of Ca­(II). Target binding to S100A12 has been shown to take place via hinge and helix 4.^30,31^ The observed pH resistance toward conformational fluctuations of these regions upon Ca­(II) binding may preserve consistent target recognition under variable biologically relevant sub neutral pH conditions. Overall, limited correlation within residues suggests that pH modulation of the conformational space and metal binding in the presence of Ca­(II) is executed via discretely coupled events in contrast to global conformational perturbations in the apo protein.

**Figure 6:**
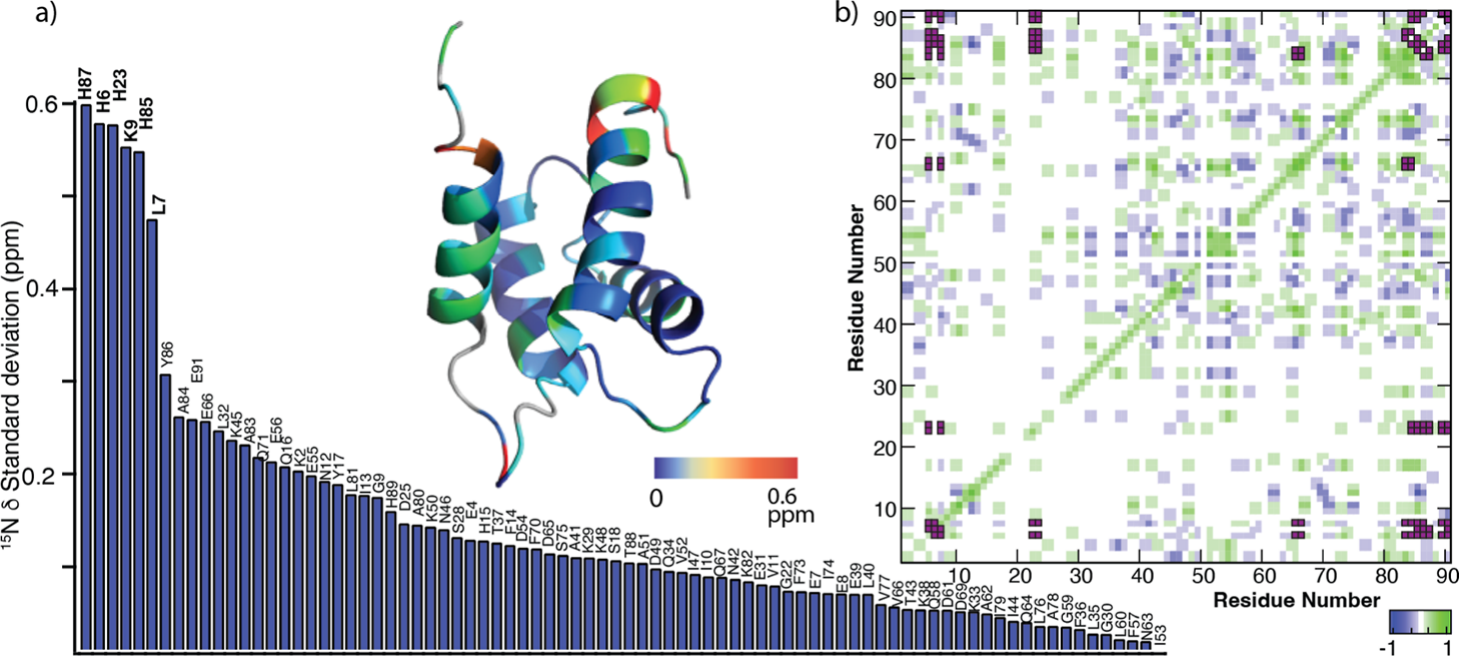
(a) Standard deviations in ^15^N^H^ chemical shifts during pH titration of Ca­(II)-S100A12 sorted in decreasing order of magnitude. (b) Residue-wise correlation between the observed chemical shift changes during the pH titration. The standard deviations in ^15^N^H^ chemical shifts are mapped on to the protein structure (only the monomeric chain is shown for the sake of clarity).

### 3.3 MD Simulations of Apo- and Ca­(II)-Bound States of S100A12

Co­(II) (Zn­(II)) binding to histidine residues takes place via the N_ε2_ atom of the histidine imidazole ring, and the loss of Co­(II) (or Zn­(II)) by S100A12 by lowering the pH could be triggered by the protonation of the histidine residues forming the His_3_Asp scaffolds. In qualitative agreement with our NMR data (vide supra) and supported by chemical arguments, this statement constitutes the basis of the following discussion and the premise for our MD simulations.

Recently, we have reported on the protonation states of all histidine residues of S100A12 at pH 6.0 and 7.0 (Table S1),^6^ demonstrating that (i) at pH 7.0, all histidine residues are neutral in both the apo- and Ca­(II)-bound states, and (ii) metal binding histidines (H15, H85, and H89) in both proteins bear protonated side chains at pH 6.0. Thus, these results suggesting that the four positively charged Ca­(II) ions do not suppress the p*K*_a_ of the His_3_Asp motif forming histidine residues exclude the possibility that electrostatics may be at the root of the acidic shift of the pH-dependent Co­(II) binding curve exhibited by the Ca­(II)-bound form of S100A12. An alternative hypothesis is that the ability of a His_3_Asp motif to withhold Co­(II) at pH 6.0 in the Ca­(II)-bound protein has a thermodynamic basis. To corroborate this argument, we carried out MD simulations to characterize the nature of the chemical bonding of the residues in the His_3_Asp motif resulting from the protonation of the metal-binding histidines.

We first focus on apo S100A12, by considering all histidine-bearing positive side chains, mimicking the protein at pH 6.0 or lower.^6^ To characterize the molecular interactions of the residues forming the His_3_Asp scaffold, we analyzed the MD trajectories and calculated the minimal distance between the atoms of the carboxyl group of D25 and either the N_δ_ or N_ε_ atoms of the metal-coordinating histidines (H15, H87, and H89). During the course of a 2 μs MD trajectory, H15 showed large fluctuations, exhibiting no meaningful interactions with D25 (Figure 7). Similar to H15, no stable close contacts were observed between H89 and D25 (data not shown). In the absence of any geometrical constraints restraining these residues, these ionizable histidine residues are susceptible to pH-induced conformational changes. This is consistent with the large pH-induced CSPs, reflecting major conformational changes for H15 (Figure 4). In the case of H89, although resonances from this residue were not observed in our NMR measurements, residues T88 and E91 in its vicinity exhibit significant CSP deviations, indicative of large conformational changes (Figure 4). With T88 and E91 undergoing major rearrangements, and in light of the long-range spatial correlations exhibited by the CSPs, it is reasonable to assume that H89 may also experience large conformational fluctuations. Thus, the predominant interaction observed in the MD simulation involving D25 is a strong salt bridge (3.0 Å) with H85 at a frequency of 0.81. Due to the relatively large conformational space available to H15 and H89, these two residues are unable to form stable salt-bridges with D25, thereby allowing for the formation of a consistent salt bridge with H85 (Figure 7). We remark that this close distance between the two oppositely charged residues H85 and D25, and the long lifetime of their contact, are both indicative of an energetically stable salt bridge bond. It is thus likely that this strong salt bridge, formed upon protonation of the metal-binding histidine residues, competes with Zn­(II)/Co­(II) binding to the His_3_Asp motif.

**Figure 7:**
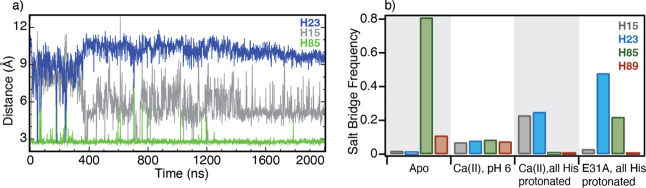
(a) Minimal distance between the O atoms of the carboxyl group of D25 and either the N_δ_ or N_ε_ atoms of H85 (green), H23 (blue), and H15 (gray) versus time, extracted from a 2 μs MD trajectory of the apo protein with all histidine side chains protonated. (b) Frequency of salt bridge formation between D25 and H15 (gray), H23 (blue), H85 (green), and H89 (red) observed in MD trajectories of apo (gray background), Ca­(II) bound mimicking pH 6.0 state (white background), Ca­(II) bound with all histidine side chains protonated (gray background), and Ca­(II)-bound E31A with all histidine side chains protonated (white background). See Methods for additional details.

In the case of Ca­(II)-S100A12, we carried out MD simulations by considering the following histidine protonation states to mimic two pH conditions. First, the metal-binding histidines (H15, H85, and H87) and H89 are protonated, whereas H23 and H6 are neutral. According to our NMR study, this set of protonation states for the histidines in the Ca­(II)-bound protein is achieved at a pH of 6.0.^6^ Second, all histidines were protonated. This situation is expected to mimic that of the protein at a pH of 4.0. MD simulations of the protein in these two protonation states show that the geometrical constraints imposed by Ca­(II) on the EF I loop hinder the formation of the D25-H85 salt bridge interaction observed in the apo protein.

In detail, MD simulations of Ca­(II)-S100A12 mimicking protonation states at pH 6.0 show the formation of this salt bridge at a dramatically reduced frequency of 0.1 compared to that of the apo protein (Figure 8), with no other significant interactions between the residues forming the His_3_Asp scaffold. Lack of stable and energetically favorable salt bridge interactions may explain the increased propensity of the His_3_Asp motif to withhold Zn­(II)/Co­(II) at pH 6.0, thus explaining the acidic shift in the Co­(II) pH titration curve shown in Figure 1. At lower pH conditions, when all histidine residues (including H23 and H6) are likely to be protonated, two intermittent salt bridge interactions are observed: (i) D25-H15 at a frequency of 0.23 and (ii) D25-H23, taking place at a frequency of 0.25. Together, these salt bridges occur at a frequency of ∼0.5 during the trajectory. Similar to the apo protein, these salt bridges are likely to compete with the ability of the His_3_Asp scaffold to chelate Zn­(II)/Co­(II). However, unlike the apo protein, in the case of the Ca­(II)-bound protein, protonation of H23 is critical for salt bridge formation. Overall, the insight provided by the MD simulations suggests that Ca­(II) to the EF I loop may serve three critical roles, which may qualitatively explain our experimental findings. First, the conformational change to the loop hinders the D25-H85 interaction. Second, structural constraints imposed on the EF I loop owing to Ca­(II) binding render (protonated) H23 and H15 amenable for salt bridge interactions with D25. Third, electrostatic repulsion by the neighboring Ca­(II) ion lowers the p*K*_a_ of H23 compared to the apo protein, thereby imparting H23 the role of a switch, delaying the onset of formation of the salt bridges and ultimately increasing the viable pH range for Zn­(II)/Co­(II) binding.

**Figure 8:**
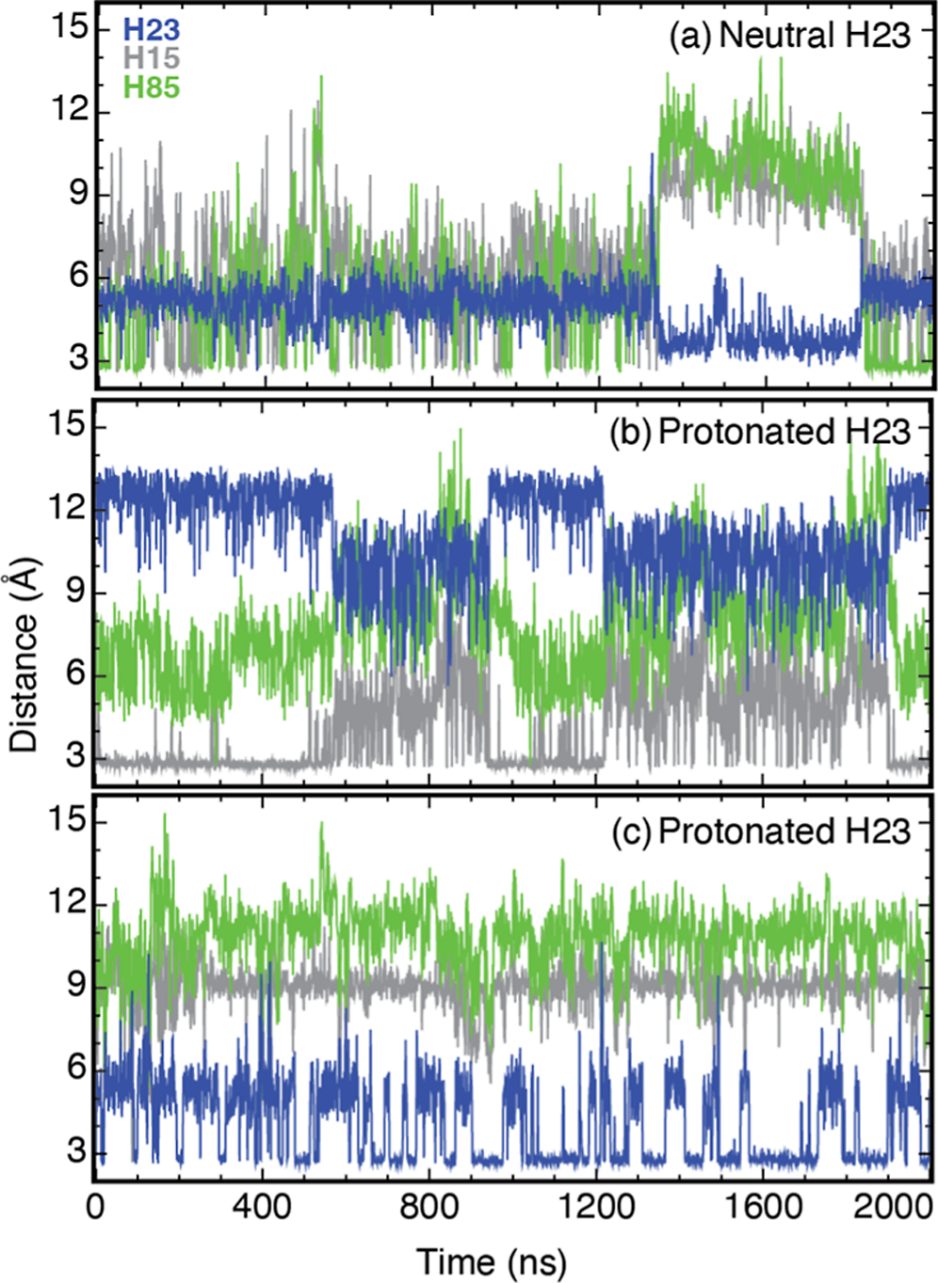
Minimal distance between the O atoms of the D25 side chain carboxyl and either the N_δ_ or N_ε_ atoms of H85 (green), H23 (blue), and H15 (gray) versus time, extracted from 2 μs long MD trajectories of the Ca­(II)-bound form of the S100A12 protein at pH 6.0 (a) and under acidic conditions when H23 is protonated, (b) and (c). The latter two panels show the distances observed in the two symmetric His_3_Asp motifs of the dimeric protein. See Methods section for additional details.

We also carried out MD simulations of the E31A mutant of S100A12, bearing Ca­(II) only in the EF-II loop. Here, all histidine residues were protonated to mimic a high proton concentration corresponding to low pH. Interestingly, these MD simulations suggest molecular interactions for the His_3_Asp motif that are intermediate between those obtained for the apo- and Ca­(II)-loaded protein. In particular, we found that, similar to the apo protein, a strong salt bridge is formed between D25 and H85 (Figure 9), albeit at a lower frequency of 0.22 (Figure 7b) compared to the apo protein. While structurally distinct, EF loops in S100 proteins are spatially coupled. The crystal structure of Ca­(II)-S100A12 shows interaction between the two loops via the formation of a short β-sheet motif, and our previous studies have demonstrated that these loops are dynamically coupled in μs-ms time scale.^19^ The existence of the D25-H85 salt bridge suggests that a lack of Ca­(II) in the EF I loop enables the formation of this unproductive interaction in the E31A variant. However, more importantly, its diminished frequency originates from the indirect modulation of the EF I loop conformation upon binding of Ca­(II) to the EF II loop. The structural influence of Ca­(II) binding to the EF II and the coupling between the loops is evident from the wild-type Ca­(II) loaded-like behavior of the variant when H23 is protonated. Conformational modulation of the EF I by the EF II loop also enables the formation of a D25-H23 salt bridge (with frequency of 0.48, Figure 7a), similar to that observed in the wild-type protein. Thus, based on these results, the observed intermediate shift in the sigmoidal pH response can be attributed to two factors. First, the presence of Ca­(II) ion in only the EF II loop of the variant suppresses the p*K*_a_ of H23, although to a lesser extent compared to the wild-type protein bearing two Ca­(II) ions in both EF loops. Second, the reduction in the frequency of the primary unproductive D25-H85 interaction, which hinders Co­(II)/Zn­(II) binding. Together, these elements could give rise to the reduced acidic shift in the pH-dependent Co­(II) binding in the E31A variant.

**Figure 9:**
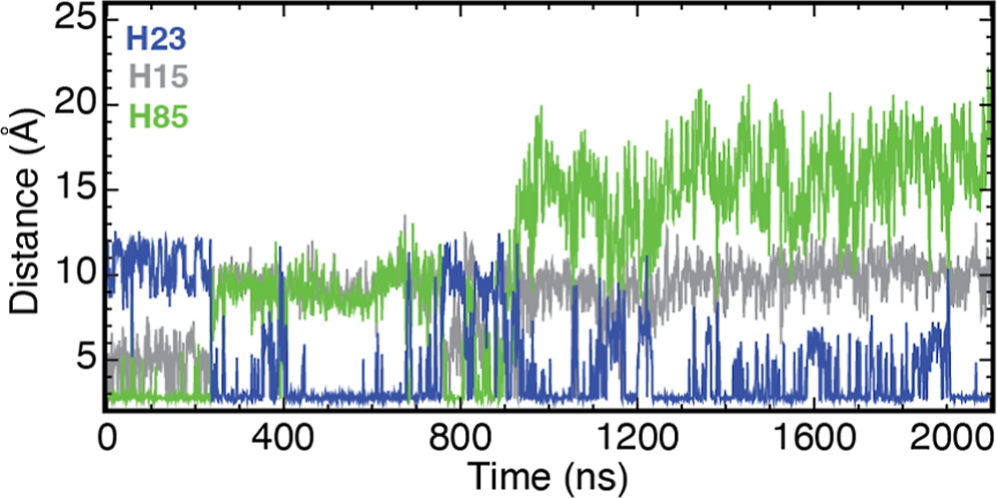
Minimal distance between the O atoms of the carboxyl group of D25 and either the N_δ_ or N_ε_ atoms of H85 (green), H23 (blue), and H15 (gray) versus time, extracted from a 2 μs long MD trajectory of the Ca­(II)-bound E31A form of S100A12 with side chains of all histidine residues protonated (mimicking low pH conditions).

## 4 Conclusions

Calcium aided restoration of Zn­(II) sequestration by S100A8/A9 and S100A12 at sub neutral pH conditions underscores its pivotal role in enabling the complex biological functions of antimicrobial human proteins. In this work, we used UV–vis and NMR spectroscopy and MD simulations to gain insight into the mechanisms underlying the role of Ca­(II) in expanding the metal chelation resistance to pH of S100A12. NMR pH titrations show that the apo protein undergoes significant conformational changes across the pH interval 4.0–9.0, which are dispersed and strongly correlated throughout the entire polypeptide. Upon Ca­(II) binding, S100A12 exhibits large pH-induced NMR CSPs confined to discrete regions. In both cases, SVD analysis of the NMR data shows that, in agreement with UV–vis titration curves, across the pH interval 4.0–9.0, the two proteins undergo several protonation events, most likely involving histidine residues switching from the neutral to the positive states upon lowering the pH. To devise a molecular picture explaining our experimental data, we carried out MD simulations of both the apo- and Ca­(II)-bound states of S100A12, with histidine residues in either the neutral or protonated states. The MD trajectories showed that, in the case of the apo protein, D25 and protonated H85 can form a strong (and long living) salt bridge, whereas in the Ca­(II)-bound protein, formation of this salt bridge is hindered by the presence of Ca­(II) in the EF loops. Only when H23 is protonated can significantly strong salt bridges be formed between D25 and H15, or between D25 and H23. These salt bridge interactions are expected to regulate Zn­(II) binding via pH and presence of Ca­(II) in the EF loops. Overall, the results suggest that Ca­(II) expands the viable pH range for metal chelation by (i) imposing geometrical constraints and thereby limiting unfavorable interactions in the His_3_Asp motif, and potentially (ii), electrostatically suppressing the p*K*_a_ of H23 (belonging to the EF I loop) and lying in close proximity to the EF II and His_3_Asp motif. Taken together, these results provide a basis for the mechanism of the regulation of S100 metal sequestration by calcium under extracellularly relevant pH conditions. The synergistic coupling between the EF loops allows these proteins to withstand sub neutral pH conditions and restore their functionality.

## Supplementary Material



## Notes

Protonation states of histidine residues in apo- and Ca­(II)-bound S100A12 at pH 6.0 and simulations of Co­(II) pH titration data assuming single proton transfer (PDF)
