# Supplementary material for: Molecular Basis of Calcium-Induced Acidic Shift in Antimicrobial Zinc Sequestration by S100A12
Source: J Phys Chem B. 2025 Sep 19;129(39):9929–38. doi: 10.1021/acs.jpcb.5c04464 (PMC12498495; doi:10.1021/acs.jpcb.5c04464)
Supplement: Supplementary file 1 [file jp5c04464_si_001.pdf]

Supporting Information for

Molecular Basis of Calcium Induced Acidic Shift in Antimicrobial Zinc Sequestration by S100A12

Mahil Kothalawala[‡,⊥], Shaan Shirazi[‡], Qian Wang[‡], Ahava Collado[‡], Angelo Bongiorno[‡,⊥,*], and Rupal Gupta[‡,⊥,*]

[‡]*Department of Chemistry, College of Staten Island, City University of New York, New York, 10314, United States*

[⊥]*Ph.D. Programs in Biochemistry and Chemistry, The Graduate Center of the City University of New York, United States*

***Corresponding author:** Rupal Gupta, Department of Chemistry, College of Staten Island, The City University of New York, USA; Angelo Bongiorno, Department of Chemistry, College of Staten Island, The City University of New York, USA

Table S1. Tautomeric states of histidine residues in S100A12 at pH 6.0.[b]

Residue	Apo	Ca(II) Bound
H6	protonated	$N^{\varepsilon 2}H$ tautomer
H15	protonated	protonated
H23	protonated	N/A[a]
H85	protonated	protonated
H87	protonated	protonated
H89	protonated	protonated

a: cannot be determined
b: from Wang, Q.; Aleshintsev, A.; Jose, A. N.; Aramini, J. M.; Gupta, R. *ChemBioChem* **2020**, *21* (9), 1372-1382

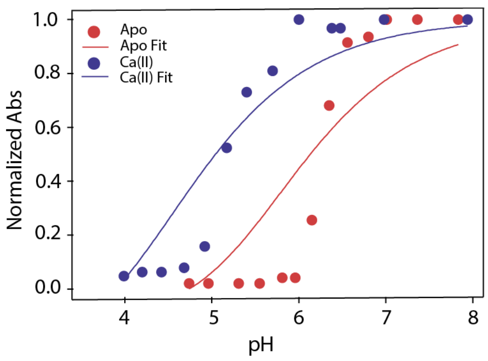

Figure S1. pH dependence of Co(II) binding to apo(•) and Ca(II) bound(•) S100A12. The solid traces are fit to experimental data assuming titration of a single proton.
